# Environment-dependent pleiotropic effects of mutations on the maximum growth rate *r* and carrying capacity *K* of population growth

**DOI:** 10.1371/journal.pbio.3000121

**Published:** 2019-01-25

**Authors:** Xinzhu Wei, Jianzhi Zhang

**Affiliations:** Department of Ecology and Evolutionary Biology, University of Michigan, Ann Arbor, Michigan, United States of America; Biological Research Center, HUNGARY

## Abstract

Maximum growth rate per individual (*r*) and carrying capacity (*K*) are key life-history traits that together characterize the density-dependent population growth and therefore are crucial parameters of many ecological and evolutionary theories such as *r*/*K* selection. Although *r* and *K* are generally thought to correlate inversely, both *r*/*K* tradeoffs and trade-ups have been observed. Nonetheless, neither the conditions under which each of these relationships occur nor the causes of these relationships are fully understood. Here, we address these questions using yeast as a model system. We estimated *r* and *K* using the growth curves of over 7,000 yeast recombinants in nine environments and found that the *r*–*K* correlation among genotypes changes from 0.53 to −0.52 with the rise of environment quality, measured by the mean *r* of all genotypes in the environment. We respectively mapped quantitative trait loci (QTLs) for *r* and *K* in each environment. Many QTLs simultaneously influence *r* and *K*, but the directions of their effects are environment dependent such that QTLs tend to show concordant effects on the two traits in poor environments but antagonistic effects in rich environments. We propose that these contrasting trends are generated by the relative impacts of two factors—the tradeoff between the speed and efficiency of ATP production and the energetic cost of cell maintenance relative to reproduction—and demonstrate an agreement between model predictions and empirical observations. These results reveal and explain the complex environment dependency of the *r*–*K* relationship, which bears on many ecological and evolutionary phenomena and has biomedical implications.

## Abstract 2

Mutations simultaneously influence the maximum growth rate r and carrying capacity K of a population, but the impacts can be concordant or antagonistic depending on the environment; this study uses data from thousands of yeast recombinants to reveal complex r-K relationships and the underlying causes.

## Author summary

Two parameters are widely used to describe density-dependent population growth: the maximum growth rate per individual (*r*) and the maximum population size or carrying capacity (*K*). The relationship between these parameters is the subject of many fundamental theories and debates in ecology and evolutionary biology. Interestingly, although only *r*/*K* tradeoffs are expected and explained thus far, *r*/*K* trade-ups have also been reported. The present study surveyed the relationship between *r* and *K* using the growth curves of over 7,000 yeast strains in nine environments, discovering that the *r*–*K* correlation changes from trade-ups to tradeoffs as the environment quality improves. Analysis of the genetic underpinnings of variations in *r* and *K* confirms that the same mutation tends to have concordant influences on *r* and *K* in poor environments but antagonistic influences in rich environments. It is proposed that the environment-dependent pleiotropic effects of mutations on *r* and *K* are a result of the tradeoff between the speed and efficiency of energy production and the energetic cost of cell maintenance relative to reproduction. The varied relation between *r* and *K* may have biomedical implications for the antibiotic control of microbial infections and the population growth of tumor cells.

## Introduction

Density-dependent population growth is commonly described by a logistic curve with two parameters: *r* and *K*. The carrying capacity *K* is the maximum population size that can be supported by the available resource in a local environment, whereas the maximum growth rate *r* is the number of individuals produced per individual per unit time when the population size is much smaller than *K*. Evolutionary biologists typically treat *r* as a measure of fitness, whereas ecologists often regard *K* as a fitness proxy [1]. Because of such biological importance of *r* and *K*, their relationship has been studied for over half a century, most often in the context of *r*/*K* tradeoffs and *r*/*K* selection [1]. Specifically, it has been argued that in fluctuating environments, population sizes are usually much lower than *K*, so increasing *K* has little effect on population growth; selection is thus focused on *r* as a means to expanding the population. Under this condition, organisms are said to be under *r* selection to become *r* strategists, which are characterized by a relatively high fecundity but low probability of surviving to adulthood, along with other traits such as small body size, early maturity onset, short generation time, and the ability to disperse offspring widely. By contrast, when the environment is more or less stable or predictable, populations often approach the carrying capacity, making raising *r* irrelevant; hence, selection is centered on *K* to increase the population size. Under this condition, organisms are said to be subject to *K* selection to become *K* strategists, which are characterized by a relatively low fecundity but high survivorship, along with a large body size, long life expectancy, and the production of fewer offspring, which often require extensive parental care until they mature [2–4]. Comparing *r*-selected and *K*-selected organisms revealed an apparent *r*/*K* tradeoff, possibly because investing energy/resources in improving *r* compromises the investment in improving *K* and vice versa [4], but it could also be because *r*-selected organisms have relatively unimpressive *K* and vice versa.

The *r*/*K* selection and *r*/*K* tradeoff were once highly fashionable topics in ecology, but they lost popularity in the 1990s when empirical studies obtained more complex results than theoretical predictions [5]. Nonetheless, the essence of *r*/*K* selection was later blended into other life-history models [6]. Studying *r*/*K* selection and *r*/*K* tradeoff with evolutionary ecology approaches can be difficult because (i) the mechanistic basis of the tradeoff is unclear, (ii) the initial environment where the relevant traits evolved is usually unknown, (iii) the natural environment is hard to manipulate, and (iv) the number of replicates/species is insufficient most of the time [5]. The topic of *r*/*K* tradeoffs was, however, revived in microbial studies in the last decade [7–11]. Although these studies have the benefits of manipulated environments and sufficient replicates, they are small in terms of genotype and environment numbers, and the *r*/*K* tradeoff is not consistently observed across experiments [7–11]. For example, a recent study reported positive correlations between *r* and *K* in bacteria and fungi across environments [11]. However, it is generally unknown under what conditions *r*/*K* tradeoffs and trade-ups, respectively, are expected. Related to this question is a lack of clear understanding of the mechanistic basis of various *r*–*K* relationships. The compromise between ATP production rate (i.e., number of ATPs generated per unit time) and efficiency (i.e., number of ATPs generated per unit resource) is commonly used to explain the *r*/*K* tradeoff [12–14], but this cannot be the whole story because it cannot explain the *r*/*K* trade-up.

Given the long history of studying the *r*–*K* relationship, it is surprising that this relationship at the mutational level is rarely researched [11]. In fact, Charlesworth showed almost 30 years ago that pure phenotypic correlations among life-history variables are unlikely to provide useful information on tradeoffs because selection and environmental effects may generate positive correlations between traits even when they have negative underlying correlations, and he suggested that studying genetic correlations can help understand evolutionarily relevant tradeoffs and predict evolutionary responses to new selective pressures [15]. In this study, we take advantage of a recently released dataset of >7,000 yeast genotypes with known genome sequences and growth curves under multiple environments to address the following suite of questions. First, do mutations simultaneously influence *r* and *K*? Second, when a mutation simultaneously influences *r* and *K*, are the effects concordant or antagonistic? Third, are the answers to the above two questions influenced by the environment, and how? Fourth, what is the mechanistic basis of the potentially varying *r*–*K* relationship? We report that the pleiotropic effects of mutations on *r* and *K* tend to be concordant under poor environments but antagonistic under favorable environments and demonstrate that these general trends are explainable by the relative impacts of two factors: the tradeoff between the speed and efficiency of ATP production and the energetic cost of cell maintenance relative to reproduction.

## Results

### Estimating *r* and *K* by fitting yeast growth data to logistic curves

Illingworth and colleagues sequenced the genomes of 85 *MAT*a and 86 *MAT*α haploid *Saccharomyces cerevisiae* strains derived from a 12th-generation two-parent intercross pool constructed from a North American strain and a West African strain that diverged from each other at 0.53% of genomic nucleotide positions [16]. Hallin and colleagues then mated each of the *MAT*a strains with each of the *MAT*α strains to obtain 7,310 diploids with known genotypes [17]. They grew these strains in nine different solid media (**S1 Table**) with four replicates and measured the cell number in each replicate by colony scan-o-matic [18] from 0 and 72 h of growth at 20 min intervals.

**Fig 1:**
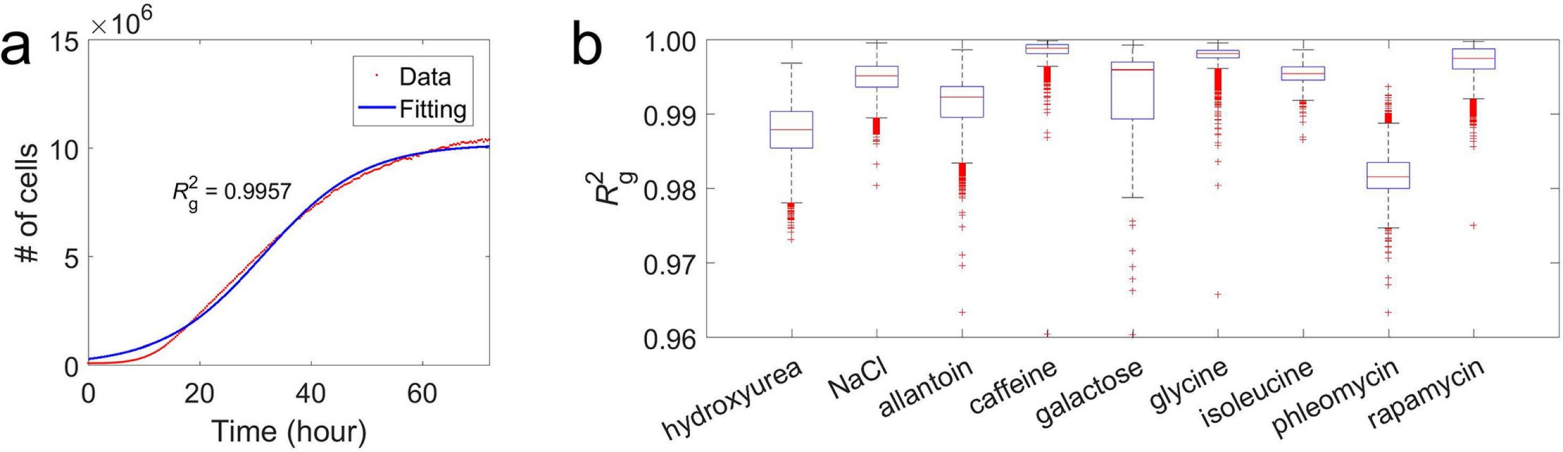
Yeast growths fit logistic curves. (**a**) An example of yeast growth in the galactose medium and the fitted logistic curve. Red dots represent observed data from one replicate of a genotype, and the blue line is the fitted curve. *R*_g_^2^ from the logistic curve is presented. (**b**) Distribution of *R*_g_^2^ among all genotypes in each of the nine environments. The lower and upper edges of a box represent qu_1_ and qu_3_, respectively; the horizontal red line inside the box indicates md; the whiskers extend to the most extreme values inside inner fences, md ± 1.5 (qu_3_–qu_1_); and the red crosses represent values outside the inner fences (outliers). Data are available at https://github.com/AprilWei001/Environment-dependent-r-K-relations. md, median; qu, quartile.

We first developed a method to simultaneously estimate the maximum growth rate *r* (number of cells produced per cell per h) and the carrying capacity *K* (number of cells) by fitting growth data to logistic curves (see Materials and methods). For each genotype under each environment, we used this method to estimate *r* and *K* for each replicate (see **Fig 1A** for an example) and then averaged among replicates that pass our quality standard (**S1 Data**); we similarly averaged the coefficient of determination (*R*_g_^2^; the subscript g refers to growth) of the fitted logistic curve among qualified replicates (see Materials and methods). We found that yeast growths tightly follow logistic curves. Across the nine environments, the median *R*_g_^2^ among genotypes is in the range of 0.979–1.000 (**Fig 1B**). Except for one environment (phleomycin), at least 75% of genotypes have *R*_g_^2^ > 0.98 (**Fig 1B**).

### Reducing environment quality turns *r*/*K* tradeoffs into trade-ups

**Fig 2:**
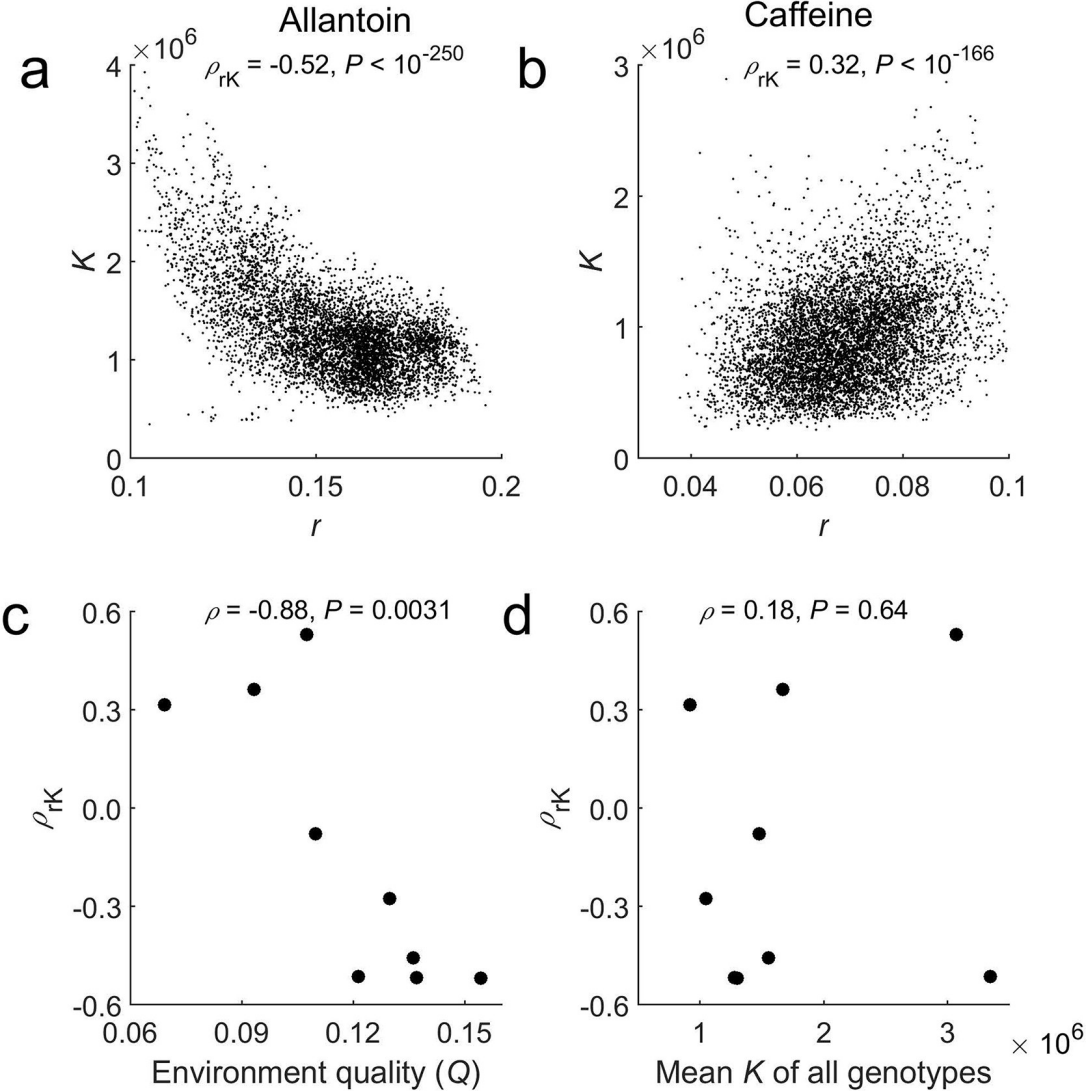
The *r*–*K* correlation among genotypes varies with environment. (**a**) An overall negative *r*–*K* correlation is observed in the allantoin medium. (**b**) An overall positive *r*–*K* correlation is observed in the caffeine medium. In (a) and (b), each dot is a genotype. The rank correlation (*ρ*_rK_) between *r* and *K* among all genotypes and the associated *P*-value are presented. (**c**) *ρ*_rK_ decreases with environment quality *Q*, which is the mean *r* of all genotypes in the environment. (**d**) *ρ*_rK_ is not significantly correlated with the mean *K* of all genotypes in the environment. In (c) and (d), each dot represents one environment. The among-environment rank correlation (*ρ*) between *ρ*_rK_ and either *Q* or mean *K* are presented. Data are available at https://github.com/AprilWei001/Environment-dependent-r-K-relations.

Under each environment, we measured Spearman's rank correlation (*ρ*_rK_) between the estimated *r* and *K* among all genotypes. In six of the nine environments, *ρ*_rK_ is significantly negative (all *P* < 10^−11^), revealing *r*/*K* tradeoffs (**S1 Fig**). But in the other three environments (NaCl, caffeine, and galactose), *ρ*_rK_ is significantly positive (all *P* < 10^−166^), showing *r*/*K* trade-ups (**S1 Fig**). For example, *ρ*_rK_ = −0.52 in the allantoin medium (**Fig 2A**) but 0.32 in the caffeine medium (**Fig 2B**). Because the same genotypes were used in all environments, the above results indicate that the environment affects the relationship between *r* and *K*. To exclude the possibility that these results are caused by biased estimations of *r* and *K*, we conducted a computer simulation in which the specified *r* and *K* are uncorrelated. The simulated data resembled the empirical data in all other aspects such as numbers of replicates, genotypes, environments, time points measured during growth, the range of *r*, the range of *K*, and *R*_g_^2^ (see Materials and methods). The simulated data were analyzed as the actual data, but in none of the environments did we find *ρ*_rK_ to differ significantly from 0. Furthermore, the estimated *r* and *K* are sufficiently accurate when compared with the values specified in the simulation (see Materials and methods). We also confirmed by computer simulation that growth need not reach saturation for reliable estimations of *r* and *K* (see Materials and methods). These results support that the observed varying *ρ*_rK_ across environments is genuine.

The type of stress does not seem to determine whether *ρ*_rK_ is positive or negative because the three environments with a positive *ρ*_rK_ belong to three different types of stress (**S1 Table**). To investigate what environmental factors impact the sign and magnitude of *ρ*_rK_, we considered environment quality *Q*, which is the mean *r* of all genotypes in the environment [19]. We found that *Q* and *ρ*_rK_ are strongly negatively correlated (*ρ* = −0.88, *P* = 3.1 × 10^−3^; **Fig 2C**), suggesting that reducing the environment quality turns *r*/*K* tradeoffs into trade-ups. As a comparison, we also calculated the mean *K* of all genotypes in an environment but found it uncorrelated with *ρ*_rK_ (*ρ* = 0.18, *P* = 0.64; **Fig 2D**). This is probably because the total amount of carbon and nitrogen provided varies among the media (**S1 Table**), making the mean *K* not directly comparable among the nine environments.

### Among-genotype variations of *r* and *K* have common genetic components

**Fig 3:**
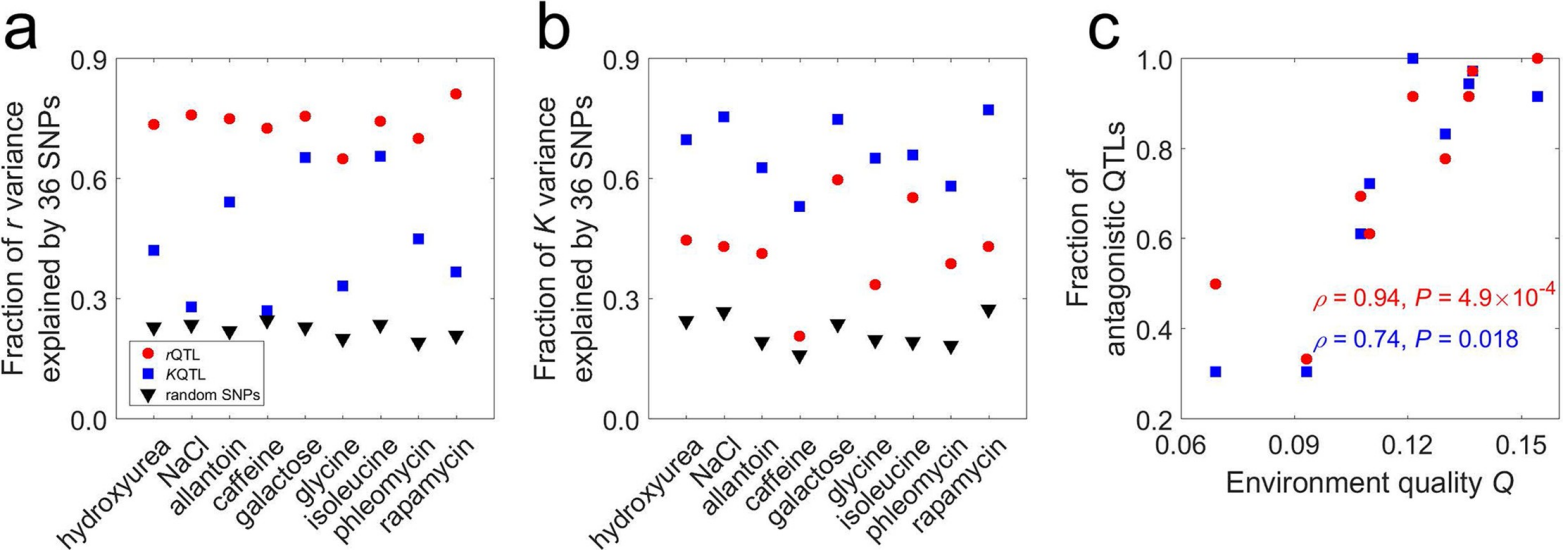
Among-genotype variations of *r* and *K* in each environment share genetic components. (**a**) Fractions of *r* variance among genotypes explainable by 36 *r*QTLs, 36 *K*QTLs, and 36 random SNPs, respectively. (**b**) Fractions of *K* variance among genotypes explainable by 36 *r*QTLs, 36 *K*QTLs, and 36 random SNPs, respectively. (**c**) Fractions of *r*QTLs and *K*QTLs that show opposite effects on *r* and *K*, respectively, increase with environment quality *Q*. Rank correlations (*ρ*) between these fractions and *Q*, as well as the associated *P*-values, are presented. Data are available at https://github.com/AprilWei001/Environment-dependent-r-K-relations. QTL, quantitative trait locus; SNP, single-nucleotide polymorphism.

To understand the genetic basis of the *r*/*K* tradeoffs and trade-ups, we respectively mapped quantitative trait loci (QTLs) for *r* (*r*QTLs) and *K* (*K*QTLs) in each environment and identified 93–96 QTLs per trait per environment (see Materials and methods). Through a series of steps that maximize the difference between the total phenotypic variance of a trait explained by QTLs and that explained by the same number of random single-nucleotide polymorphisms (SNPs), we retained the 36 most significant QTLs per trait in each environment (**S2 Data**) for further analysis (see Materials and methods). In each environment, the 36 top *r*QTLs together explain 65%–81% of the total variance of *r* (**Fig 3A**) as well as 21%–60% of the total variance of *K* in the same environment (**Fig 3B**). Similarly, the 36 top *K*QTLs together explain 53%–77% of the total variance of *K* (**Fig 3B**) as well as 27%–66% of the total variance of *r* in the same environment (**Fig 3A**). That *r*QTLs partially explain the *K* variance and vice versa has two possible explanations. First, some *r*QTLs and *K*QTLs share the same underlying causal mutations. In other words, some mutations are pleiotropic, affecting both *r* and *K*. Second, *r*QTLs and *K*QTLs have distinct causal mutations and are independently distributed in the genome, but *r*QTLs explain the *K* variance and vice versa owing to the linkage disequilibrium between *r*QTLs and *K*QTLs in the mapping population, which could still exist after 12 generations of crosses. Under this explanation, 36 randomly picked SNPs should explain the *r* variance as much as the 36 *K*QTLs do. But what we found is that in each of the nine environments, the 36 *K*QTLs explain the *r* variance much better than the 36 randomly chosen SNPs do (*P* < 0.001 based on 1,000 random samplings of 36 SNPs) (**Fig 3A**). The same is true when comparing *r*QTLs and random SNPs in explaining the *K* variance (**Fig 3B**). These observations refute the second potential explanation, suggesting that some *r*QTLs and *K*QTLs share causal mutations, which is supported by the recent finding that altering the ribosomal RNA gene copy number in *Escherichia coli* simultaneously alters *r* and *K* [11].

### Fraction of antagonistic QTLs rises with environment quality

That the sign of *ρ*_rK_ turns from positive into negative as the environment quality *Q* rises (**Fig 2C**) and that *r* and *K* share underlying genetic components (**Fig 3A** and **3B**) predict that the fraction of QTLs with antagonistic effects on *r* and *K* rises with *Q*. To confirm this prediction, in each environment, we estimated the effects of each *r*QTL on *r* and *K* by regression (see Materials and methods). If the two effects are of the same direction, the QTL has concordant effects; otherwise, it has antagonistic effects. In seven of the nine environments, most *r*QTLs show antagonistic effects; in one other environment (the NaCl medium), most *r*QTLs show concordant effects. In the remaining environment (the caffeine medium), equal numbers of *r*QTLs show concordant and antagonistic effects. The fraction of antagonistic *r*QTLs indeed rises with *Q* (*ρ* = 0.94, *P* = 4.9 × 10^−4^; **Fig 3C**).

We similarly analyzed the effects of *K*QTLs on *r* and *K* in each environment. In seven of the nine environments, most *K*QTLs exhibit antagonistic effects. The opposite is true in the remaining two environments (the NaCl and caffeine media). Again, the fraction of antagonistic *K*QTLs rises with *Q* (*ρ* = 0.74, *P* = 0.018; **Fig 3C**). Not unexpectedly, neither the fraction of antagonistic *r*QTLs nor the fraction of antagonistic *K*QTLs in an environment correlates significantly with the mean *K* of all genotypes in the environment (*P* > 0.5 in both cases).

### Environment-dependent pleiotropic effects of individual QTLs on *r* and *K*

**Fig 4:**
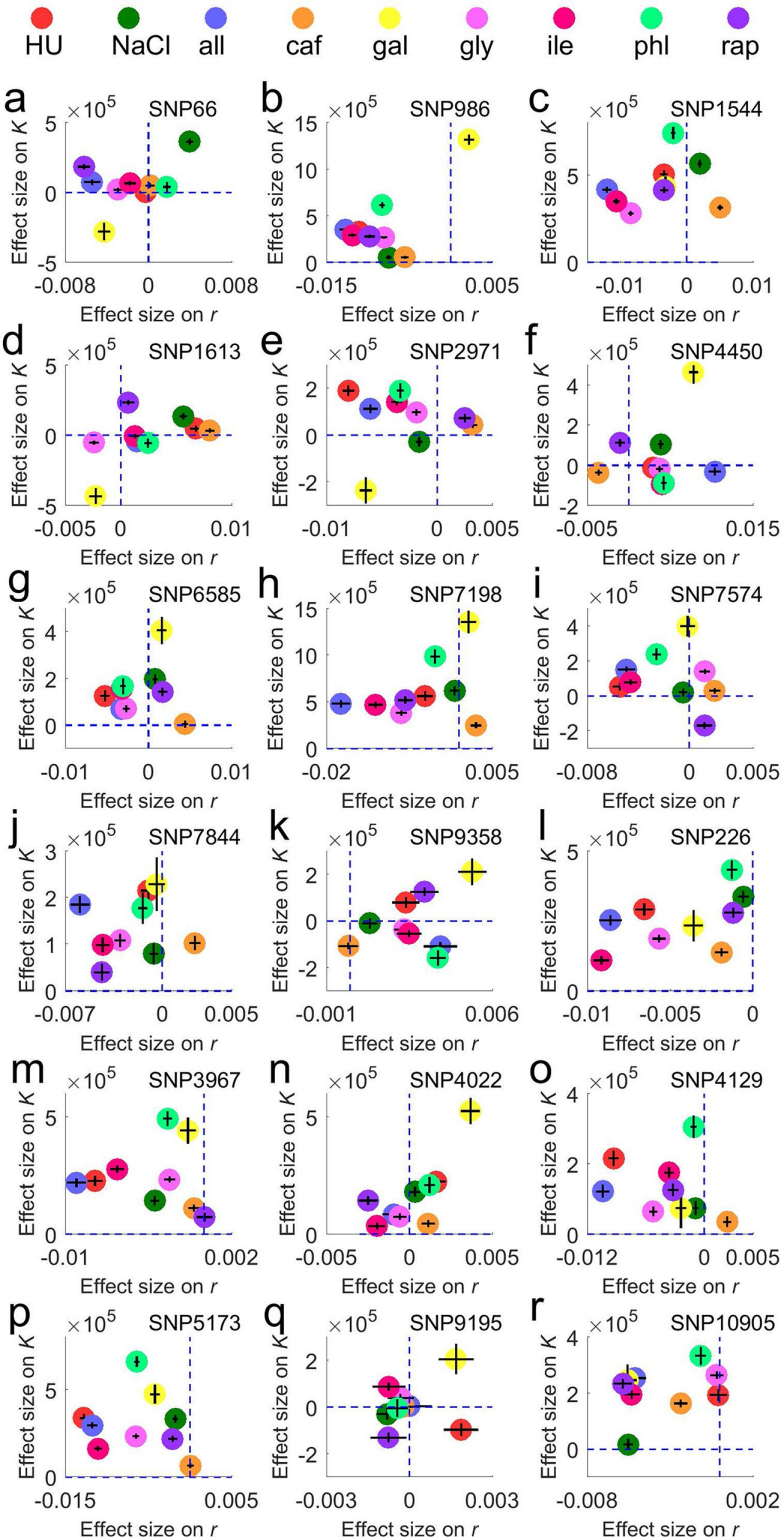
Representative SNPs of individual QTLs show varying pleiotropic effects on *r* and *K* in different environments. (**a**)–(**r**) Each panel is the result for one QTL, with the representative SNP labeled at the right corner. The *x*-axis shows the effect of the North American allele at the SNP on *r*, and the *y*-axis shows the effect of the same allele on *K*. Blue dashed lines indicate zero effects. The effects in each environment are shown by the central position of a sphere with the corresponding color, estimated by the difference in mean phenotypic value between homozygotes with the North American allele and homozygotes with the West African allele, and the SEs of the effects are shown by error bars. Data are available at https://github.com/AprilWei001/Environment-dependent-r-K-relations. all, allantoin; caf, caffeine; gal, galactose; gly, glycine; HU, hydroxyurea; ile, isoleucine; NaCl, sodium chloride; phl, phleomycin; QTL, quantitative trait locus; rap, rapamycin; SE, standard error; SNP, single-nucleotide polymorphism.

The above results strongly suggest that the phenotypic effects of a given QTL on *r* and *K* may be antagonistic in one environment but concordant in another. In other words, the environment modulates the type of pleiotropy of the QTL, which we refer to as pleiotropy by environment interaction, a form of genotype by environment interaction [20]. To our knowledge, QTL pleiotropy by environment interaction has not been reported beyond one case in plants [21]. To explore this phenomenon in our data, we examined each 3 kb genomic segment—which harbors 1.5 genes and 3.0 mapping SNPs on average—and counted the number of times that an *r*QTL or *K*QTL identified in an environment resides in this segment. This treatment is necessary because (i) the causal genetic variant of a QTL cannot be traced to the nucleotide resolution despite much of the linkage in the original parental strains being broken in 12 generations of crosses and (ii) each mapping SNP may represent multiple SNPs that are in complete linkage disequilibrium (see Materials and methods). We considered only the 36 top *r*QTLs and 36 top *K*QTLs per environment. We referred to a segment as an enriched segment if four or more QTLs were found in the segment among the nine environments. A total of 21 enriched segments were detected. By contrast, our simulation showed that only 0.83 segments are expected to have ≥4 QTLs if all 36 × 2 × 9 = 648 QTLs are randomly distributed in the yeast genome. Among the 21 segments, 18 harbor at least one *r*QTL and at least one *K*QTL. Because one segment is expected to have only 0.144 QTLs if all QTLs have distinct causal mutations, the ≥4 QTLs in each of these 18 segments likely have the same causal mutation. Because the causal mutation is unknown, an SNP representing the causal mutation was chosen (see Materials and methods), and its effects on *r* and *K* in each environment were estimated. **Fig 4** shows the effects of these 18 representative SNPs on *r* and *K* in each of the nine environments, and they clearly demonstrate pleiotropy by environment interactions. For example, SNP #66 has significant concordant effects on *r* and *K* in the NaCl and galactose media but significant antagonistic effects in the rapamycin, allantoin, and isoleucine media (**Fig 4A**).

### Why do *r*/*K* tradeoffs turn into trade-ups as *Q* lowers?

The prevailing explanation of the *r*/*K* tradeoff is the compromise between ATP production rate and efficiency, which states that increasing the rate of ATP production per unit time improves the growth rate but reduces the efficiency of resource utilization by lowering the total amount of ATP produced, causing *K* to decrease [12–14]. This model, however, cannot explain why lowering *Q* turns *r*/*K* tradeoffs into trade-ups, as observed in our study. One deficiency of the model is the implicit assumption that the amount of ATP used per generation is independent of the growth rate. Population growth requires energy for producing new cells as well as energy for maintaining existing cells. While the per-generation cost for the former is probably independent of the growth rate, the cost for the latter should be proportional to the generation time *T*, which equals ln2/*r*_N_, where *r*_N_ (≤ *r*) is the growth rate when the population size is *N*. Indeed, as early as 50 years ago, Prit showed in multiple organisms that the extra substrates (glucose or glycerol) needed to produce the same amount of dry weight increases linearly with the inverse of the growth rate [22]. Hence, it is possible that when *r* is low, increasing *r* raises *K* because the per-generation cell-maintenance cost is reduced in spite of a lowered efficiency in resource utilization [23]. Below, we examine this model quantitatively.

Let *a* be the per-cell maintenance cost of energy per h. Hence, the per-cell maintenance cost per generation is *aT* = *aln*2/*r*_N_, where *T* is the generation time in h. Let *b* be the energy cost to produce a new cell. Thus, the total energy cost per cell per generation is *aln*2/*r*_N_ + *b*, and the corresponding cost per cell per h is *a* + *br*_N_/*ln*2. The above result indicates that as *r*_N_ increases, the energy used and produced per h, or ATP production rate, must increase. The tradeoff between ATP production rate and efficiency dictates that the efficiency of resource usage, *f*(*r*_N_), must then decline. Hence, *f*(*r*_N_), which is between 0 and 1, is a decreasing function of *r*_N_. Let the amount of resource usage per cell per generation be *C*_N_ when the population size is *N*. Following a recent study [24], we have

$$C_{N}f\left(r_{N}\right)=aln2/r_{N}+b.$$ (1)

Let us now consider the situation of *N* << *K*, under which *r*_N_ = *r* and *C*_N_ = *C*. So, Eq 1 can be written as

C=(aln2/r+b)/f(r). (2)

It is difficult to derive an analytical formula relating *r* and *K* from Eqs 1 or 2 because the exact form of *f*(*r*_N_) is unknown and because *C*_N_ changes with population growth as a result of changes of *r*_N_ and *f*(*r*_N_). Nevertheless, when the total amount of resource is fixed, the larger the *C* or *C*_N_, the fewer generations the population can grow for, and hence, the smaller the *K*.

Taking derivatives on both sides of Eq 2, we get

$$\frac{dC}{dr}=\frac{-\frac{aln2f\left(r\right)}{r^{2}}-f^{\prime }\left(r\right)\left(\frac{aln2}{r}+b\right)}{{\left[f\left(r\right)\right]}^{2}}=\frac{\left[-aln2f\left(r\right)]+[-f^{\prime }\left(r\right)\left(arln2+br^{2}\right)\right]}{{\left[f\left(r\right)\right]}^{2}r^{2}}.$$ (3)

On the right-hand side of Eq 3, the denominator is positive, the first term of the numerator is negative, and the second term of the numerator is positive. Hence, d*C*/d*r* may be positive or negative, depending on the values of *a*, *b*, and *r* and the function *f*(*r*). Now let us consider the scenario when *r* approaches 0. Given that *f*(*r*) will approach 1, *f* ′(*r*) cannot be infinity. Hence, the second term of the numerator in the right-hand side of Eq 3 approaches 0, while the first term of the numerator remains considerably negative. Consequently, d*C*/d*r* < 0, meaning that *C* decreases with *r*, which results in *r*/*K* trade-ups. Let us turn to the scenario when *r* is very large. Now, the first term of the numerator is negligible relative to the second term, leading to d*C*/d*r* > 0 and *r*/*K* tradeoffs. In other words, regardless of *a*, *b*, and *f*(*r*), the model creates *r*/*K* trade-ups at very low *r* and tradeoffs at very high *r*.

To analyze the behavior of the model further, especially when *r* is not too small nor too large, we assume that *f*(*r*) = 1 − (*r*/*r*_MAX_)^*w*^, where *r*_MAX_ is the maximum possible *r* of any genotype in any environment and *w* > 0. Based on the finding that the cost of maintenance per h is about 1% of the cost of reproduction in yeast [24], we assume *a* = 0.01 and *b* = 1. We drew the numerical relationship between *C* and *r* when *r*_MAX_ = 0.5 and *w* = 3 (**S2 Fig**). One can see that *C* declines as *r* increases to an intermediate value (approximately 0.13) and then rises as *r* further increases. Hence, *K* should rise and then decline as *r* increases, creating *r*/*K* trade-ups when *r* is small but tradeoffs when *r* is large.

The above finding is made without specifying how *r* is altered. Hence, it applies when *r* is altered by an environmental shift, a mutation, or a combination of the two, as long as the parameters of the model stay more or less unchanged and *K* is measured under a fixed amount of resource when different environments are compared. Because *Q* is defined by the average *r* across all genotypes, it follows that an overall *r*/*K* trade-up among genotypes is observed in low-*Q* environments, while an overall tradeoff is observed in high-*Q* environments (**Fig 2C**). Lipson proposed verbally that when maintenance cost is considered, *r*/*K* trade-ups should be observed in slow-growth environments and tradeoffs should be observed in fast-growth environments [23]. His proposal is supported by observations from our model and empirical data.

**Fig 5:**
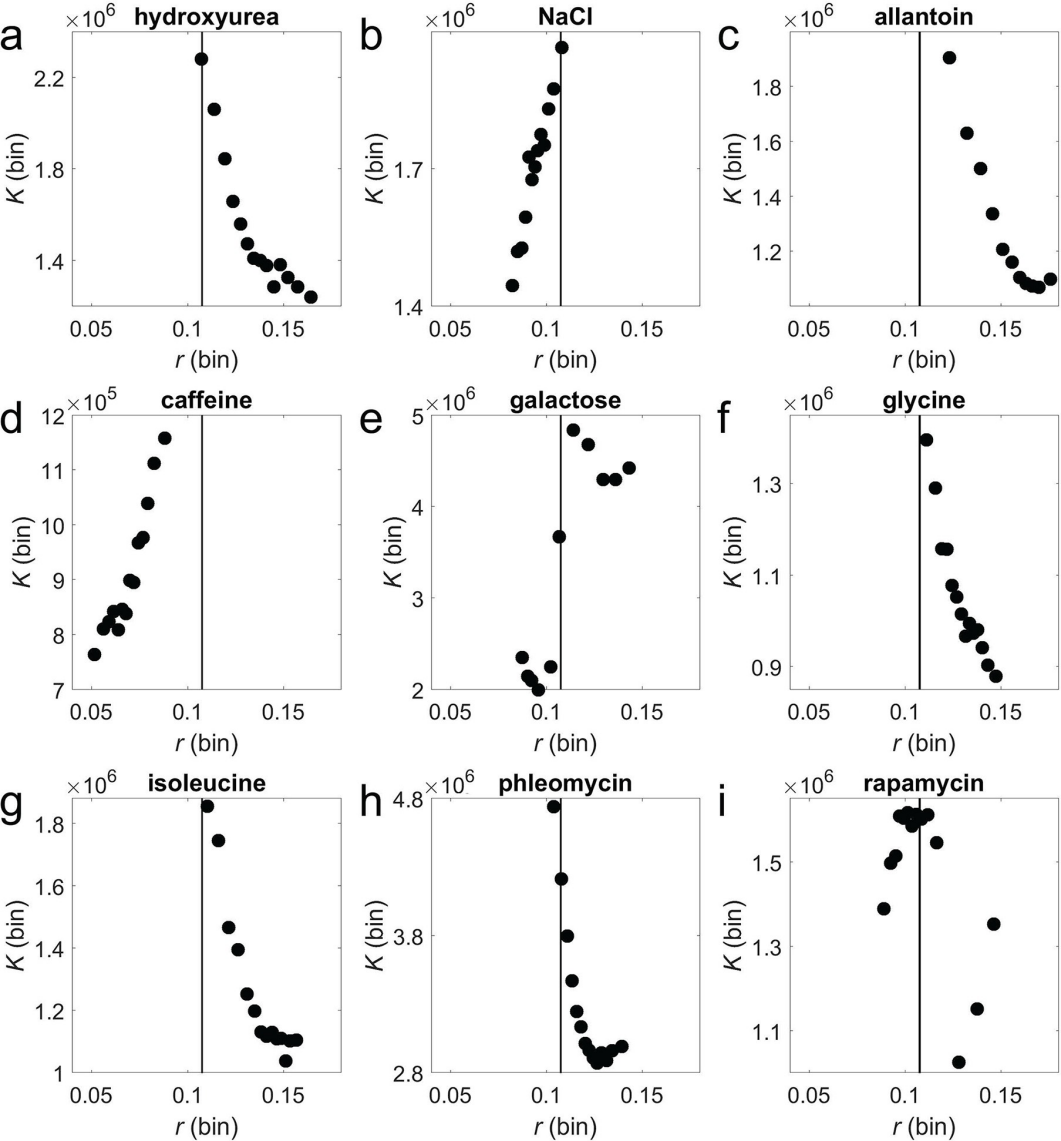
Varying *r*–*K* relationships among genotypes across different ranges of *r* in each of the nine environments. (**a**)–(**i**) Each panel shows *r*–*K* relationships in one environment labeled on the top of the panel. Each dot shows the average *r* and average *K* from a bin of 500 genotypes grouped by *r*. The same vertical black line is shown on all panels, the position of which is determined by the average *r* of the group with the highest *K* among the nine environments. Note that the *y*-axes of different panels are not directly comparable because of the variation in the amount of resource among media. Data are available at https://github.com/AprilWei001/Environment-dependent-r-K-relations.

Furthermore, our model predicts that, under a given environment, *ρ*_rK_ for a subgroup of genotypes could be positive or negative depending on the range of *r* for this subgroup of genotypes. In other words, it is possible to observe a positive *ρ*_rK_ for a subgroup of low-*r* genotypes and a negative *ρ*_rK_ for another subgroup of high-*r* genotypes in the same environment, provided that the range of *r* among genotypes in the environment is large enough. In addition, our model predicts that the critical *r* value at which *r*/*K* tradeoffs turn into *r*/*K* trade-ups should be more or less the same in different environments if *a*, *b*, and *f*(*r*) are similar among different environments. To verify these predictions, in each environment, we divided all genotypes into bins of 500 genotypes based on their *r* values in the environment. We then computed the mean *K* and mean *r* of each bin. In each environment, we identified the bin with the highest mean *K* and then averaged the mean *r* of this bin across the nine environments, which arrived at *r*_tp_ = 0.1076 (the subscript "tp" stands for turning point; see black vertical line in **Fig 5**). We found that in most but not all environments, *K* tends to increase with *r* when *r* < *r*_tp_ but decrease with *r* when *r > r*_tp_, even when the *r* range spans *r*_tp_ in an environment (**Fig 5**). Thus, the *r*/*K* tradeoffs and trade-ups can simultaneously appear in one environment, and the turning point between tradeoffs to trade-ups is similar among the nine environments.

Admittedly, there may be other models that could explain the *r*–*K* trade-up. For example, a mutation that renders some strains more efficient in using a nutrient than other strains can result in an *r*–*K* trade-up. But this hypothesis cannot explain why the *r*–*K* trade-up turns into tradeoff when *Q* increases because the ability to better use a nutrient could occur in both high- and low-*Q* environments. By contrast, the maintenance cost coupled with the conflict between the speed and efficiency of ATP production can explain trade-ups, tradeoffs, and the turn from trade-ups into tradeoffs when *Q* rises. Although we cannot prove that our model is the only possibility, it appears to be the simplest and probably the most general explanation.

## Discussion

Using the growth data of over 7,000 yeast strains in nine environments, we conducted the largest-ever investigation of the relationship between *r* and *K*. We showed an overall *r*/*K* tradeoff in high-quality environments but an overall *r*/*K* trade-up in low-quality environments, where the quality of an environment is measured by the average maximal growth rate (*r*) of all genotypes in the environment. By mapping *r*QTLs and *K*QTLs, we found that at least some mutations simultaneously influence *r* and *K*. Interestingly, the effects of the same mutation on these two traits can be concordant in one environment but antagonistic in another. In general, concordant mutational effects on *r* and *K* are more common in low-quality environments, while the opposite is true in high-quality environments. Finally, we proposed a model involving a compromise between the speed and efficiency of ATP production and the relative costs of cell maintenance and division that satisfactorily explains our observations. Our model predicts that *r*/*K* tradeoffs and trade-ups can even coexist in a single environment in different ranges of *r* values, which is subsequently confirmed by the empirical data.

Warringer and colleagues measured the growth rate and efficiency of 39 *S*. *cerevisiae* strains, 39 *S*. *paradoxus* strains, and a few strains from other yeasts in a large number of liquid media [25]. But they reported rate-efficiency tradeoffs across all strains examined in only two media. Nevertheless, we found that more media in their data show tradeoffs if only intraspecific variations are considered. For instance, we found rate-efficiency tradeoffs in 12 of the 196 media among *S*. *cerevisiae* strains and in 39 of the 196 media among *S*. *paradoxus* strains. Interestingly, the rate-efficiency correlation turns from positive into negative values as the average growth rate in a medium increases (Spearman’s *ρ* = −0.199, *P* = 0.005 in *S*. *cerevisiae*; *ρ* = −0.130, *P* = 0.070 in *S*. *paradoxus*; **S3 Fig**). Thus, our primary finding appears to hold in liquid media as well.

Recently, Reding-Roman and colleagues observed both *r*/*K* tradeoffs and trade-ups when examining microbial growths of multiple genotypes in multiple media that differ in the glucose concentration [11]. However, their findings are distinct from ours in that they observed a maximal *r* when *K* is intermediate, while we observed a maximal *K* when *r* is intermediate. Furthermore, their explanatory model is based on the Monod function [26], which neglects the cell-maintenance cost [22]. Because their observation was based on a relatively small number of genotypes and their environments varied in the concentration of only one component (glucose), the generality of their findings is unclear. At any rate, their observations differ from ours and their model cannot explain our observations.

While the classic *r*/*K*-selection theory predicts that selecting for *r* leads to a reduction in *K* and vice versa, our findings paint a more complex picture. In a constant environment, adaptation will likely improve *r* and *K* concordantly if the initial *r* is low. But when *r* reaches a certain level, further adaptation will cause antagonistic changes of *r* and *K*. Whether *r* or *K* will further increase while the other trait will decrease depends on which of the two traits is the main target of selection. These predictions can be tested using Lenski's long-term experimental evolution of 12 populations of *E*. *coli* in a constant low-glucose medium. Novak and colleagues examined the relationship between *r* and *K* in the first 20,000 generations of evolution of these *E*. *coli* populations [7]. Their results are broadly consistent with our predictions. For instance, they reported that both *r* and *K* increased quickly in the first 2,000 generations, after which *r* continued to improve slowly, but *K* stopped rising and even declined in some populations. Apparently, *r* is the main target of selection in this case. While Novak and colleagues reported no clear correlation between *r* and *K* among the 12 populations at the end of 20,000 generations, their Fig 3 showed a positive *r*–*K* correlation among populations with relatively low *r* values and a negative correlation among populations with relatively high *r* values [7]. Similarly, previous mixed reports of *r*/*K* tradeoffs and trade-ups [7–10] are actually expected rather than surprising. Hence, considering the varying intrinsic relationship between *r* and *K* is critical to predicting how *r* and *K* respond to natural selection and largely explains why Pianka’s *r*/*K*-selection–based prediction of life-history traits [4] does not always work [5].

Our findings may also have implications in medicine. For instance, our results suggest that, in applying antibiotics to control microbial infection, it is important to apply a sufficiently high dose such that *r* is below the turning point *r*_tp_. Only in this range will reducing *r* also lower *K*; otherwise, reducing *r* will increase *K*. The same principle may apply in the treatment of cancer, which is intimately related to the growth of the tumor cell population [27,28]. Nevertheless, we caution that, because our discovery is made in a unicellular organism, its generality, especially among multicellular organisms, awaits future exploration.

We identified a number of QTLs with concordant effects on *r* and *K* in one environment but antagonistic effects in another. Such pleiotropy by environment interaction means that a mutation that cannot be fixed in one environment because of antagonistic effects on two traits may be easily fixed in another environment when its effects become concordant and hence has evolutionary implications. But how common pleiotropy by environment interaction is remains unknown, although both pleiotropy [29] and genotype by environment interaction [20,30,31] appear prevalent.

Our findings illustrate the necessity and power of discerning the relationship between phenotypic traits at the mutational level for understanding the cause of their positive or negative correlation among individuals, populations, or species. With the rapid progress in genomic technology and high-throughput phenotyping, this approach promises to offer deeper and broader insights into phenotypic variation and evolution.

## Materials and methods

### Genotype and growth data of diploid yeast hybrids

We acquired from Hallin and colleagues the unsmoothed growth data of 7,310 diploids produced from all pairwise crosses between 85 *MAT*a and 86 *MATα* haploid strains of *S*. *cerevisiae* [17]. The haploids were randomly drawn from a 12th-generation two-parent intercross pool derived from a North American wild strain and a West African wild strain [17]. The colony size for each diploid genotype was measured and cell number inferred at 217 time points from 0 to 72 h at 20 min intervals with four replicates by scan-o-matic, a high-resolution automatic microbial growth phenotyping approach [18]. The four replicates were initiated from different precultures and run in different instruments and plate positions in the scanner to minimize bias. Because the cell number estimation is based on colony scan, the estimated *K* reflects the total volume of the cell population and is robust to cell size. The diploids were grown in nine different solid agar media, which are synthetic complete media with additional stressors or alternative carbon or nitrogen source (allantoin, caffeine, galactose, glycine, hydroxyurea, isoleucine, NaCl, phleomycin, and rapamycin) (**S1 Table**). Because the genomes of all 171 haploids were sequenced [16], all 7,310 diploids have known genome sequences [17]. Note that the original experiment contained 86 *MAT*a and 86 *MAT*α haploids, but all crosses involving one *MAT*a strain were contaminated and removed.

There are two potential biases in measuring growth from Hallin and colleagues' experiment [17]. First, the growth of a colony could be affected by its neighbors on the plate; this is referred to as the positional effect. Second, some regions on the plate may have systematically higher or lower growths because of differential lighting and evaporation of water; this is referred to as the spatial effect. Hallin and colleagues used grid reference correction [17] because the grid reference was shown to be useful in correcting the spatial effect in the original development of scan-o-matic by Zackrisson and colleagues [18]. Nevertheless, there is one distinction between Hallin and colleagues' data [17] and Zackrisson and colleagues' data [18] that could make the helpful correction in Zackrisson and colleagues' work detrimental in Hallin and colleagues' study. Specifically, the grid reference correction was verified in a plate of 1,536 colonies of the same genotype [18]; there was no positional effect on this plate because all positions had the same neighbors. In Hallin and colleagues' experiment, 384 controls of the same genotype were placed on each plate. A control colony in Hallin and colleagues was potentially subject to both the spatial effect and positional effect because different controls no longer shared the same neighbors. If a control colony grew rapidly because its neighbors grew slowly and were outcompeted by the control, this rapid growth was due to the positional effect. If one attempts to correct it by the grid reference, one is mistakenly assuming that the rapid growth is due to the spatial effect, and the correction introduces a bias, making the corrected neighboring genotypes’ growth rates even lower than the true values. Therefore, performing the grid reference correction can bias the estimation of genetic effects for the sake of correcting nongenetic effects. In addition, it is possible that *r* and *K* are differentially influenced by neighbors because *r* is determined mostly by earlier sections of a growth curve when competition among neighbors are not strong, while *K*, a feature determined mostly by later sections of a growth curve, is more likely influenced by neighbors. However, because each genotype had four replicates at different plate positions in the scanner, the spatial effect is mostly randomized and uncorrelated with the genotype. There is therefore little need to correct for the spatial effect. This said, we performed the normalization as in Hallin and colleagues and confirmed that our primary finding that *r*–*K* trade-ups turn into tradeoffs when *Q* rises still holds (Spearman’s *ρ* = −0.75, *P* = 0.026). Similarly, because different strains were placed randomly on plates, the positional effect on each strain is random so is not expected to create general trends as discovered in our analysis. Indeed, as mentioned in the Discussion, the above turn from tradeoffs into trade-ups is also present for yeast growth in liquid media, which has no spatial or positional effect.

### Estimating *r* and *K*

The logistic equation was used to describe density-dependent population growth [32], and it was popularized by Raymond Pearl and Lowell Reed when they substituted *r* and *K* into the Verhulst model [33]. As early as 1913, the logistic growth of yeast was demonstrated by Carlson [34]. Our estimation of *r* and *K* from growth data is based on the following logistic equation.

$$\frac{dN}{dt}=rN(1-\frac{N}{K})$$ (4)

Integrating Eq 4 leads to

$$N=\frac{K}{1+(\frac{K}{N_{0}}-1)e^{-rt}},$$ (5)

where *N*_0_ is the initial population size and *t* is the growth time. The *r* estimated here is also known as *r*_0_ in the literature and is the maximum cell growth rate. It should not be confused with the maximum population growth rate, often written as *r*_max_ and estimated from the mid-log phase of a growth curve.

We first estimated *r* and *K* for each replicate of each genotype in each environment by fitting Eq 5 to the data of cell number *N* and time *t* using the NonLinearModel.fit function in Matlab. We then removed low-quality replicates in the following manner. We assumed that *r* and *K* estimates that are far from the nearest neighbors are outliers and set cutoffs based on the fold difference between outliers and medians. Because *K* has a wider range than *r*, different cutoffs for *r* and *K* were used. In practice, we removed all replicates whose estimated *r* is larger than 200% or smaller than 50% of the median *r* from all *r* estimates from all genotypes in the same environment. We similarly removed all replicates whose estimated *K* is larger than 400% or smaller than 25% of the median *K* estimate from all genotypes in the same environment. The majority of removed replicates were extreme outliers, with *r* or *K* estimates being negative or hundreds of times bigger than nonoutliers. Changing the lower *r* cutoff to 33%, higher *r* cutoff to 300%, lower *K* cutoff to 20%, and higher *K* cutoff to 500% impacts <1% of the number of retained replicates. After the quality control, in each environment, 93.2%–100% of genotypes have at least three retained replicates. The *r* and *K* estimates of a genotype in an environment are the average values of all remaining replicates. For each remaining replicate, we computed the fraction of variance in the growth data explained by the logistic regression (*R*_g_^2^) and then computed the average *R*_g_^2^ across the remaining replicates. We found no correlation between mean *R*_g_^2^ across genotypes in an environment and the mean *r* or *K* of all genotypes in the environment. We calculated the standard error (SE) of the *r* and *K* estimates from replicates. The median SE of *r* among all genotypes varies from 0.0034 to 0.013 in the nine environments, while the median SE of *K* among all genotypes varies from 1.2 × 10^5^ to 2.6 × 10^5^ in the nine environments. The median SE of *r* (or *K*) is uncorrelated with mean *r* (or *K*) among environments. We also calculated the standard deviations of *r* and *K* among genotypes under each environment to be used in simulations (see below).

To exclude the possibility that the observed correlation between *r* and *K* is an artifact of our *r* and *K* estimation, we performed a computer simulation. We simulated the growth of 7,000 genotypes in nine environments to best mimic the real data. In each environment, the *r* and *K* of all genotypes used in the simulation followed normal distributions with the same means and standard deviations as estimated from the actual data. We then computed the cell number using the logistic curve from 0 to 72 h at 20 min intervals. We added a random noise to each computed cell number at each time point; the noise follows a normal distribution with mean = 0 and variance = median (1 − *R*_g_^2^) in each environment × SST (i.e., the total sum of squares of cell numbers for each replicate). By doing so, our median fitted *R*_g_^2^ from simulated data equals the empirical median *R*_g_^2^. Four independent replicate growth datasets were simulated per genotype per environment. Using the simulated data, we estimated *r* and *K* for each replicate of each genotype as in the estimation using the actual data. As expected, the *r* and *K* estimates from the simulated data have similar ranges as those from the actual data. In each simulated environment, 95.1%–99.9% of simulated genotypes have *r* and *K* estimated. Among them, 71.6%–74.6% of genotypes have estimated *r* and *K* that, respectively, deviate from the simulated value by <1%, and 93.0%–97.6% of the genotypes have estimated *r* and *K* that, respectively, deviate from the simulated value by <20%. Hence, our estimation of *r* and *K* is accurate under logistic growth. Of the nine simulated environments, none showed a significant correlation between *r* and *K* upon multiple-testing corrections. We also confirmed by computer simulation that growth need not reach saturation for reliable estimations of *r* and *K*. Specifically, we simulated growth using low *r* and high *K* to avoid saturation, resulting in a median last-hour growth rate that was 23.3% of the initial growth rate. Yet, estimates of *r* and *K* were generally accurate and unbiased.

### QTL mapping

Before QTL mapping, we first coded the genotype at each SNP as 0, 1, or 2 if it was homozygous for the West African allele, heterozygous, or homozygous for the North American allele, respectively. We then filtered the SNPs that contain redundant information such that only the middle SNP is maintained when several neighboring SNPs are in complete linkage disequilibrium. This resulted in 13,350 remaining SNPs for QTL mapping.

We mapped *r*QTLs and *K*QTLs in each environment following a recent QTL study [35], using a false discovery rate (FDR) of 0.05. Briefly, this approach performs multiple rounds of mapping. In each round, at most one most significant SNP in each chromosome will be mapped as a QTL, and the residuals from fitting all mapped QTLs from all previous rounds will be used for the next round of mapping. FDR is calculated by a permutation test. We stopped the mapping after six rounds, resulting in 93–96 QTLs per trait. We calculated the total phenotypic variance explained by all mapped QTLs (*R*^2^). We then removed the QTL that has the smallest effect on total *R*^2^ and recalculated the total *R*^2^ explained using all remaining QTLs. We repeated this process and removed small-effect QTLs one by one until we retained 48, 36, 24, or 18 QTLs per trait. By doing so, we acquired equal numbers of *r*QTLs and *K*QTLs in each environment. We also calculated the total fraction of phenotypic variance explained (*R*^2^_SNPs_) by 96, 48, 36, 24, or 18 randomly picked SNPs, respectively. When we retained 48 QTLs, the averaged fraction of *R*^2^ explained for all traits is *R*^2^_QTLs_ = 0.738. This value reduced to 0.703 when we retained 36 QTLs. The averaged *R*^2^_QTLs_ dropped quickly when fewer than 36 QTLs were considered. We found that the difference between *R*^2^_QTLs_ and *R*^2^_SNPs_ is maximized when 36 QTLs were compared with 36 random SNPs. Focusing on these 36 large-effect QTLs instead of 93–96 total QTLs per trait allowed us to study how environment affects mutational pleiotropy with increased confidence.

We performed a linear regression using the genotypes of the 36 *r*QTLs in one environment to predict the *r* in that environment. The regression coefficient for each *r*QTL was used as a measure of the effect of this *r*QTL on *r*. Similarly, a regression using the 36 *r*QTLs to predict *K* in that environment gave the effect of each *r*QTL on *K*. The same method was used to estimate the effects of each *K*QTLs on *r* and on *K*.

### The *r*–*K* relationship and the rate–yield relationship

In addition to the *r*–*K* relationship, the rate–yield relationship is frequently discussed in the literature [7,36], in which rate refers to the growth rate and yield refers to the dry weight produced per mole of substrate. The *r*–*K* relationship is equivalent to the rate–yield relationship when *K* is measured under a fixed amount of resource, because *K* = yield × amount of resource in moles.

## Supporting information

S1 Fig The among-genotype relationship between *r* and *K* in each of the nine environments. Each panel shows one environment labeled on top of the panel. Each dot represents one genotype.
(PDF)

S2 Fig Model-predicted relationship between maximum growth rate (*r*) and energy cost per cell per generation (*C*) using the following parameters: *a* = 0.01, *b* = 1, *r*_MAX_ = 0.5, *w* = 3. See main text for details. (PDF)

S3 Fig **The growth rate–yield correlation in liquid media turns from positive into negative as the average rate in a medium increases in (a) *S*. *cerevisiae* and (b) *S*. *paradoxus*.** Each dot represents one growth medium. Both the rate and yield estimates were from Warringer and colleagues [25]. The *x*-axis shows the average growth rate of all 39 measured strains in an environment, while the *y*-axis shows the rank correlation between rate and yield among the 39 strains in the same medium.
(PDF)

S1 Table Variables and stresses among the nine growth media. (PDF)

S1 Data Estimated *r* and *K* for each genotype in each of the nine environments. (XLSX)

S2 Data The top 36 *r*QTLs and top 36 *K*QTLs in each of the nine environments. QTL, quantitative trait locus.
(XLSX)

## Abbreviations

FDR: false discovery rate
QTL: quantitative trait locus
SE: standard error
SNP: single-nucleotide polymorphism
