# Supplementary figures and images for: Environment-dependent pleiotropic effects of mutations on the maximum growth rate r and carrying capacity K of population growth
Source: PLoS Biol. 2019 Jan 25;17(1):e3000121. doi: 10.1371/journal.pbio.3000121 (PMC6364931; doi:10.1371/journal.pbio.3000121)

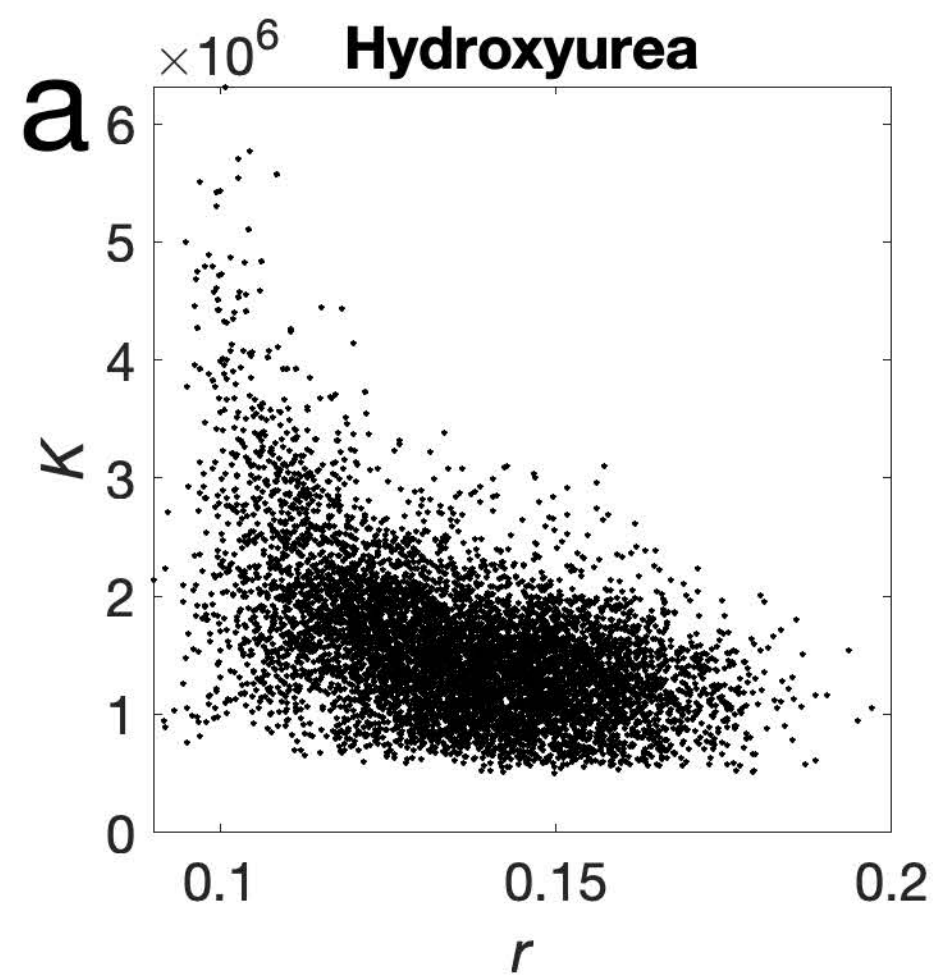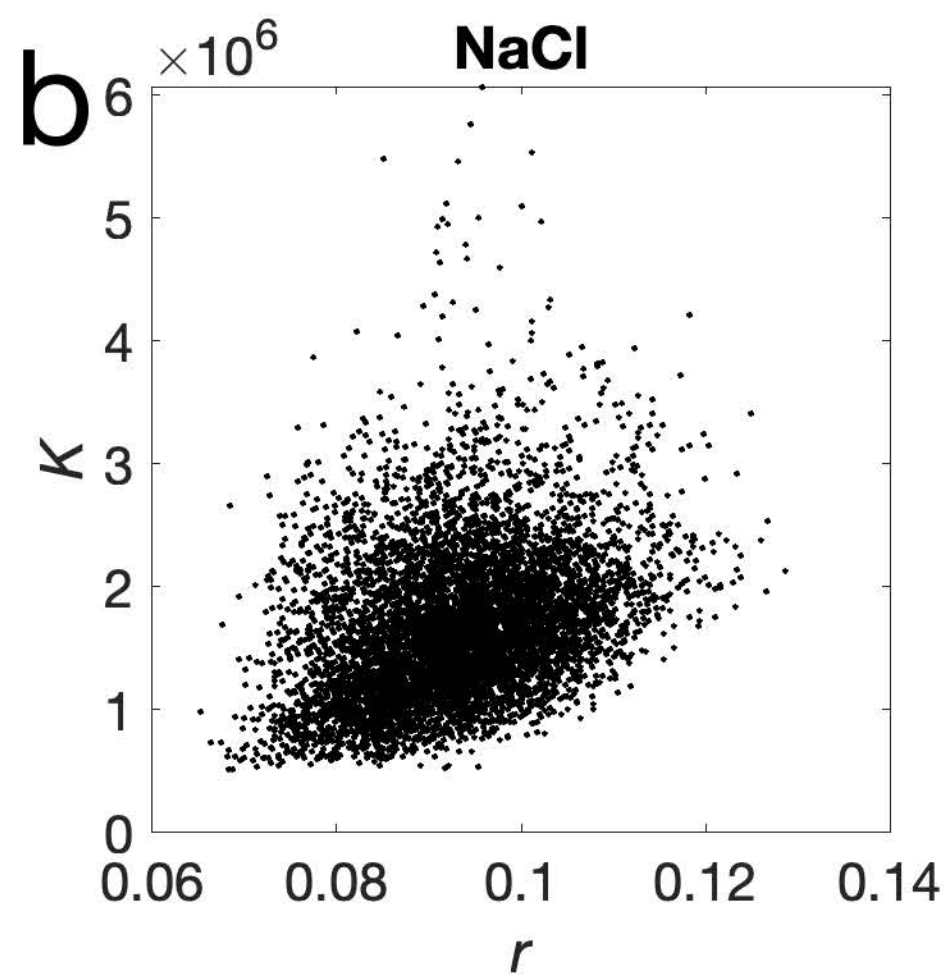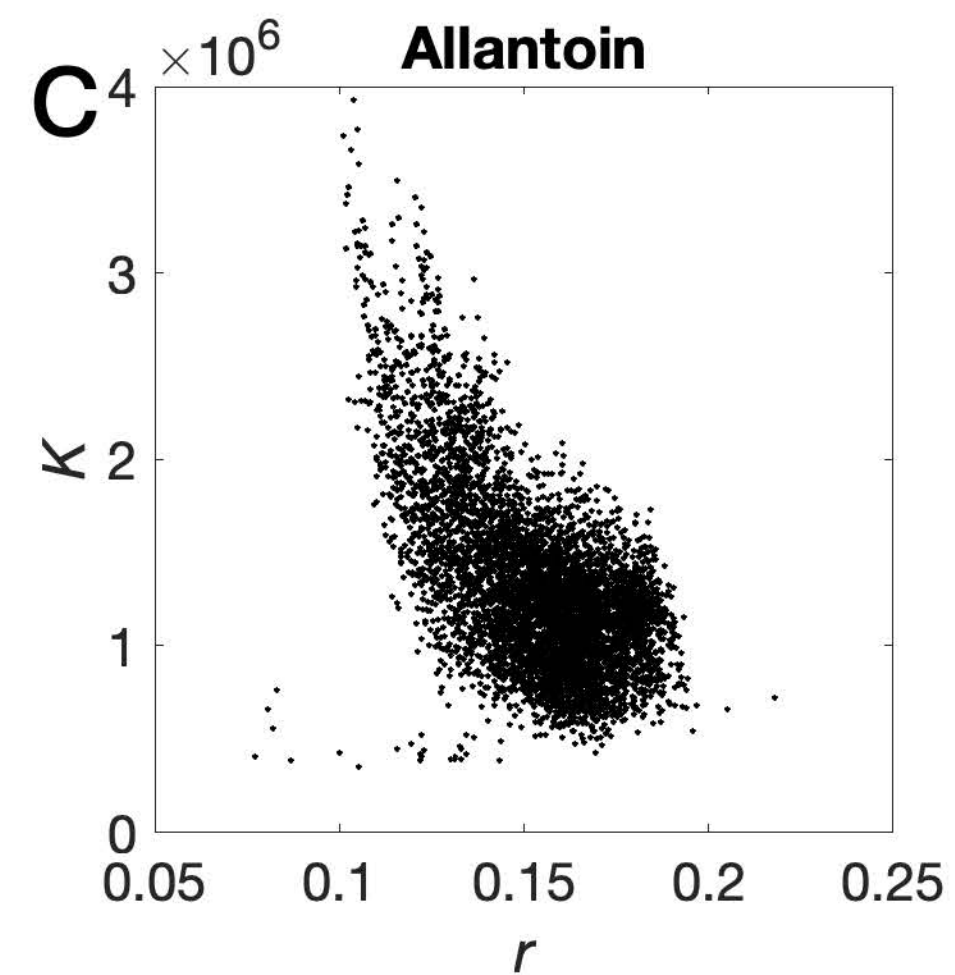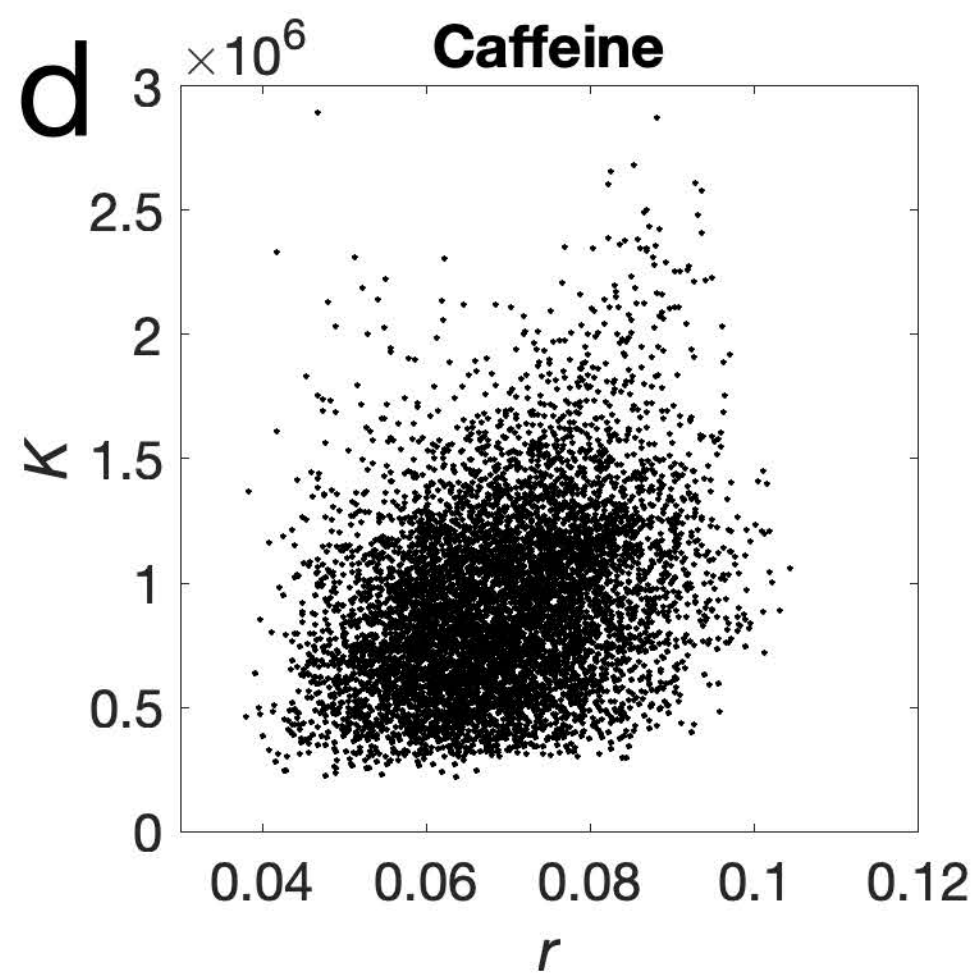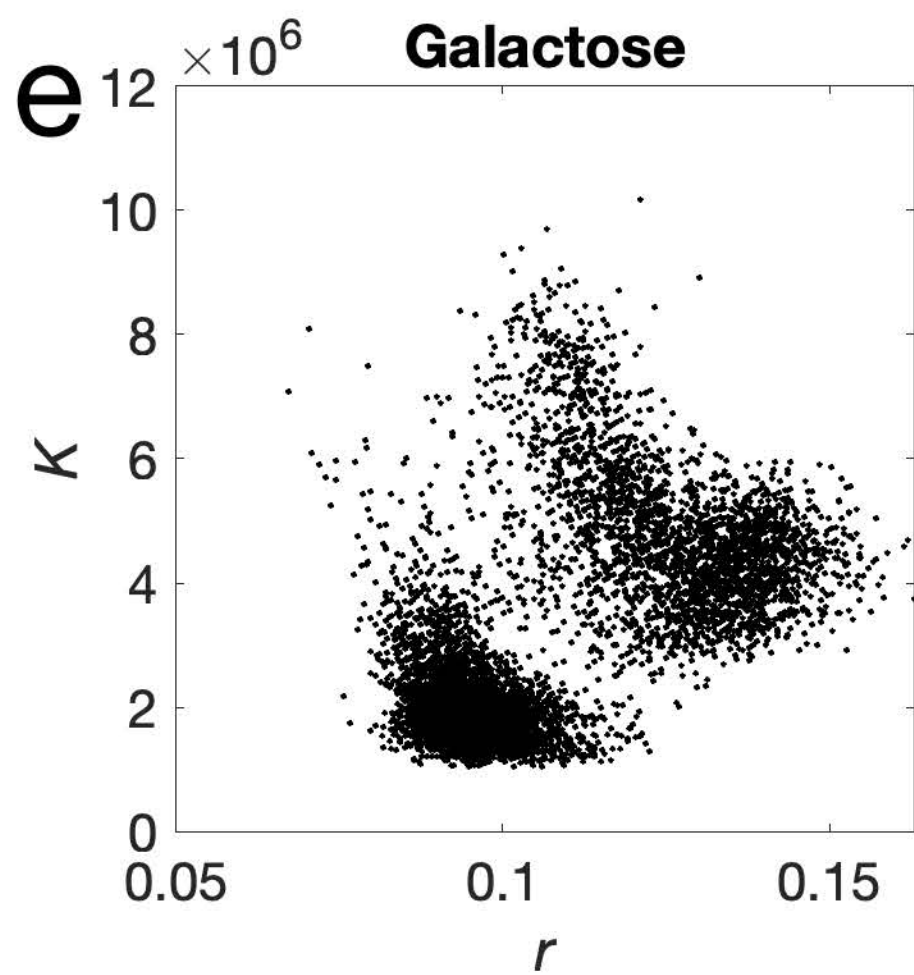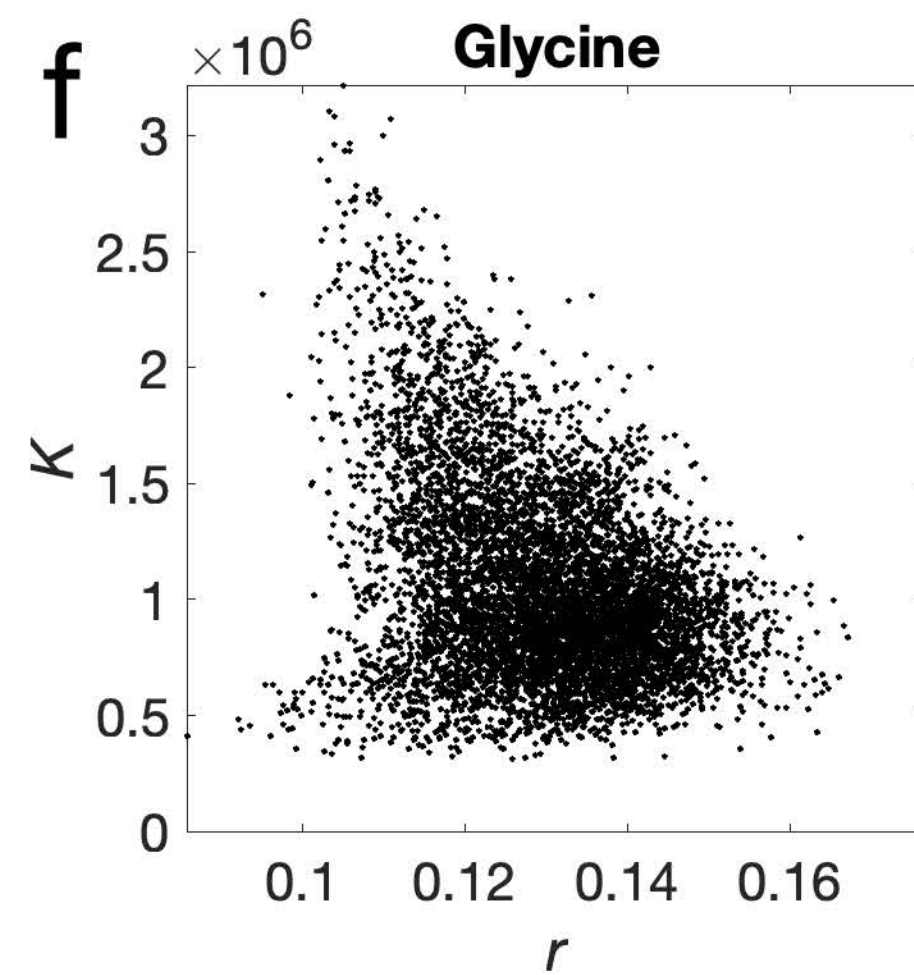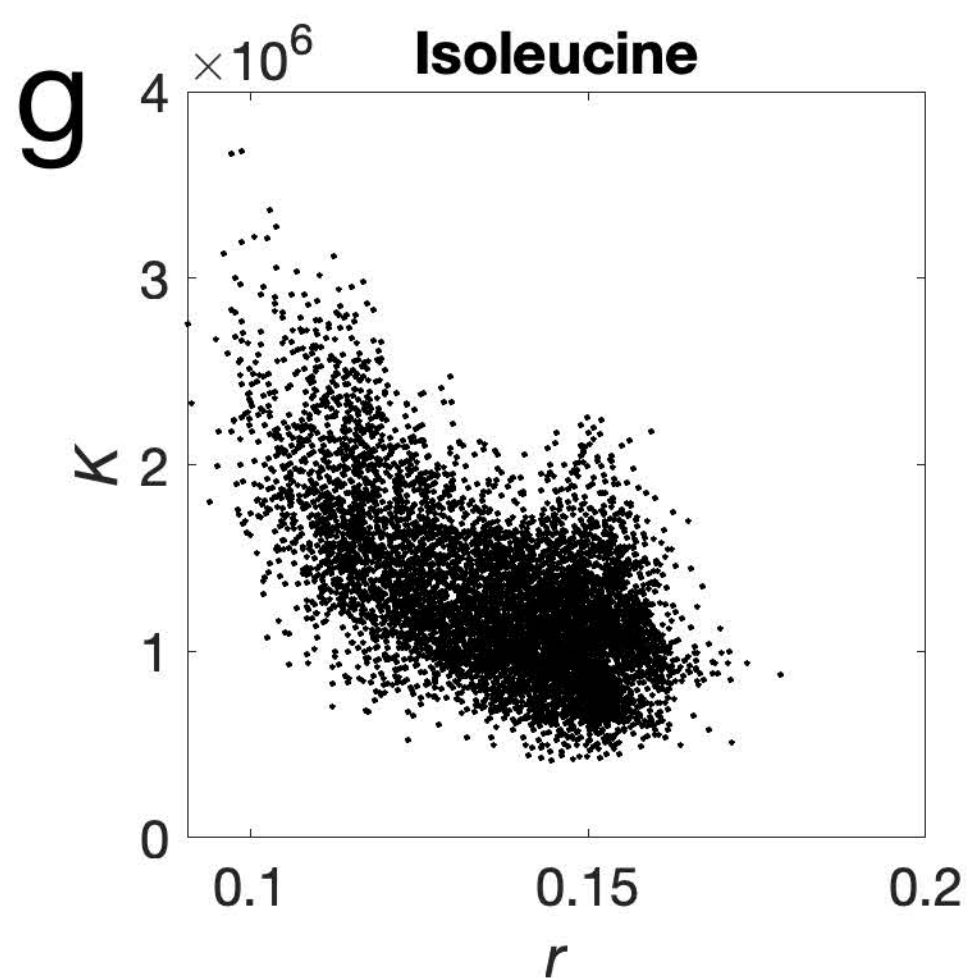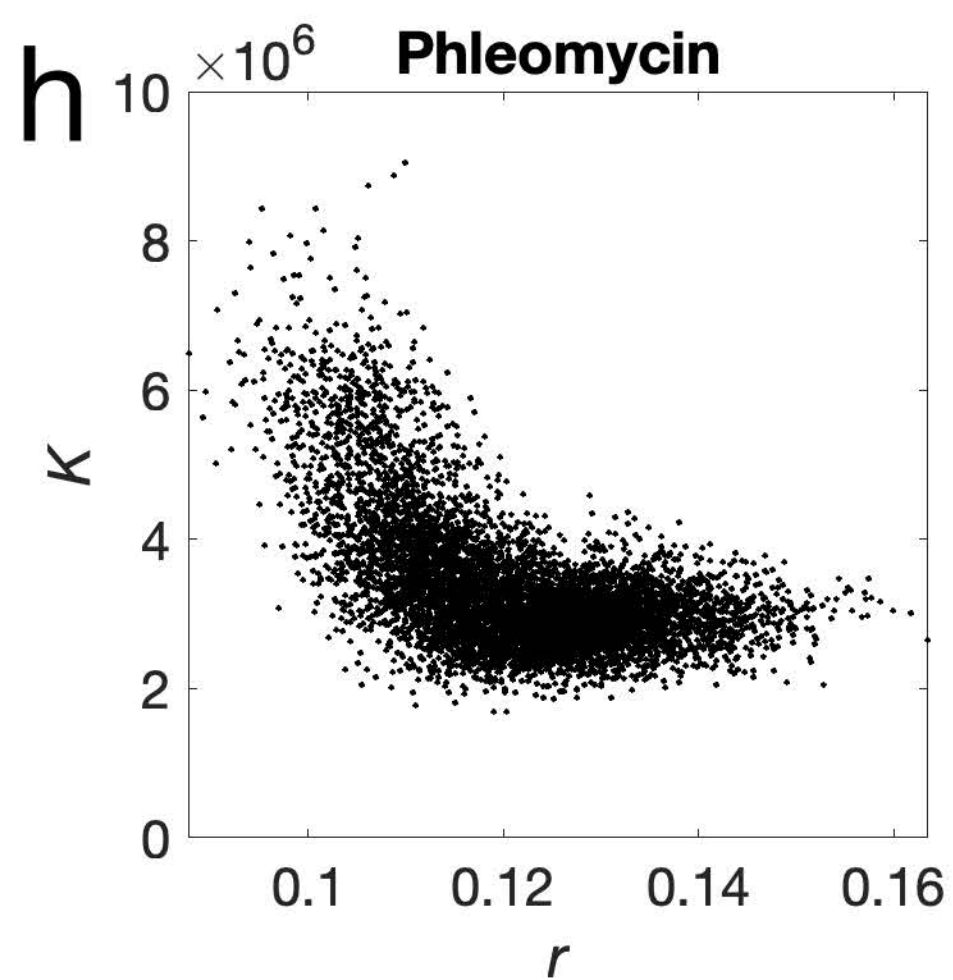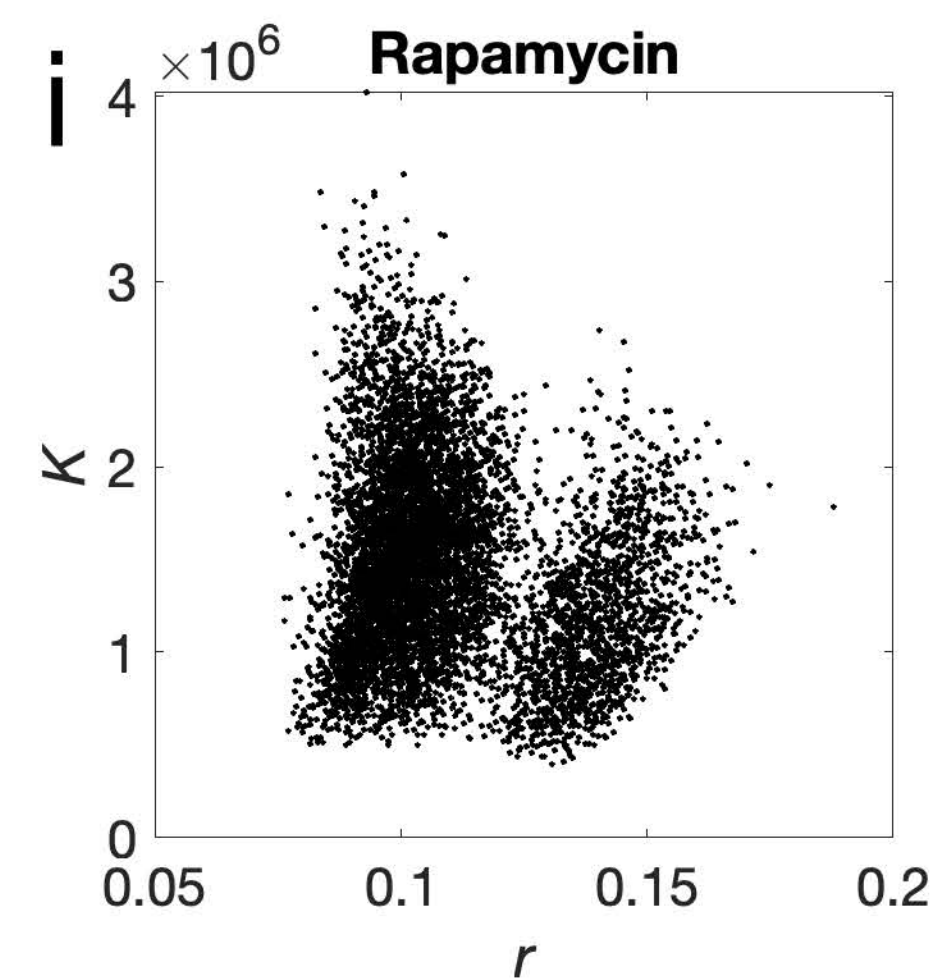

Supplement: S1 Fig — Each panel shows one environment labeled on top of the panel. Each dot represents one genotype. (PDF) [file pbio.3000121.s001.pdf]

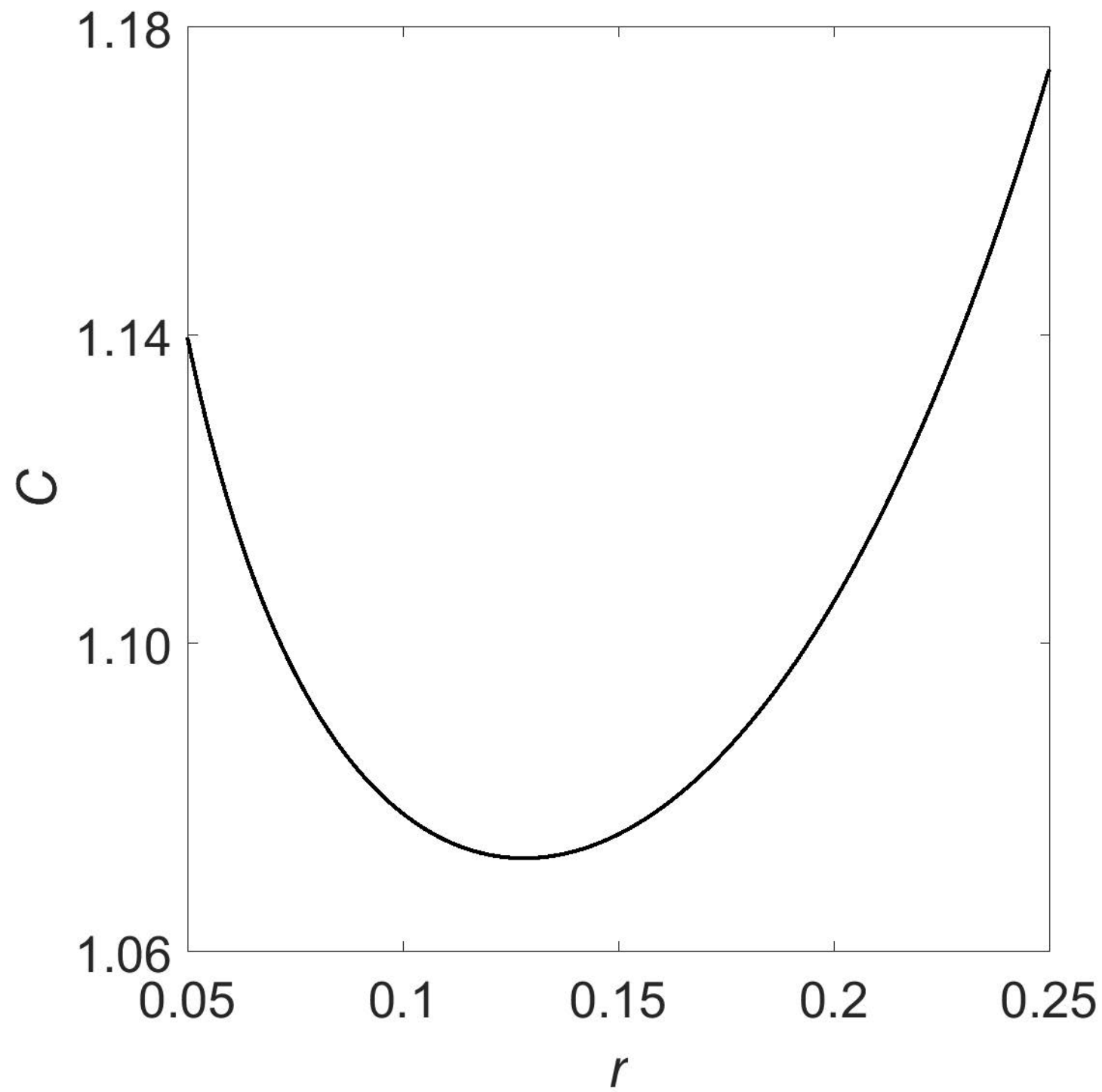

Supplement: S2 Fig — (PDF) [file pbio.3000121.s002.pdf]

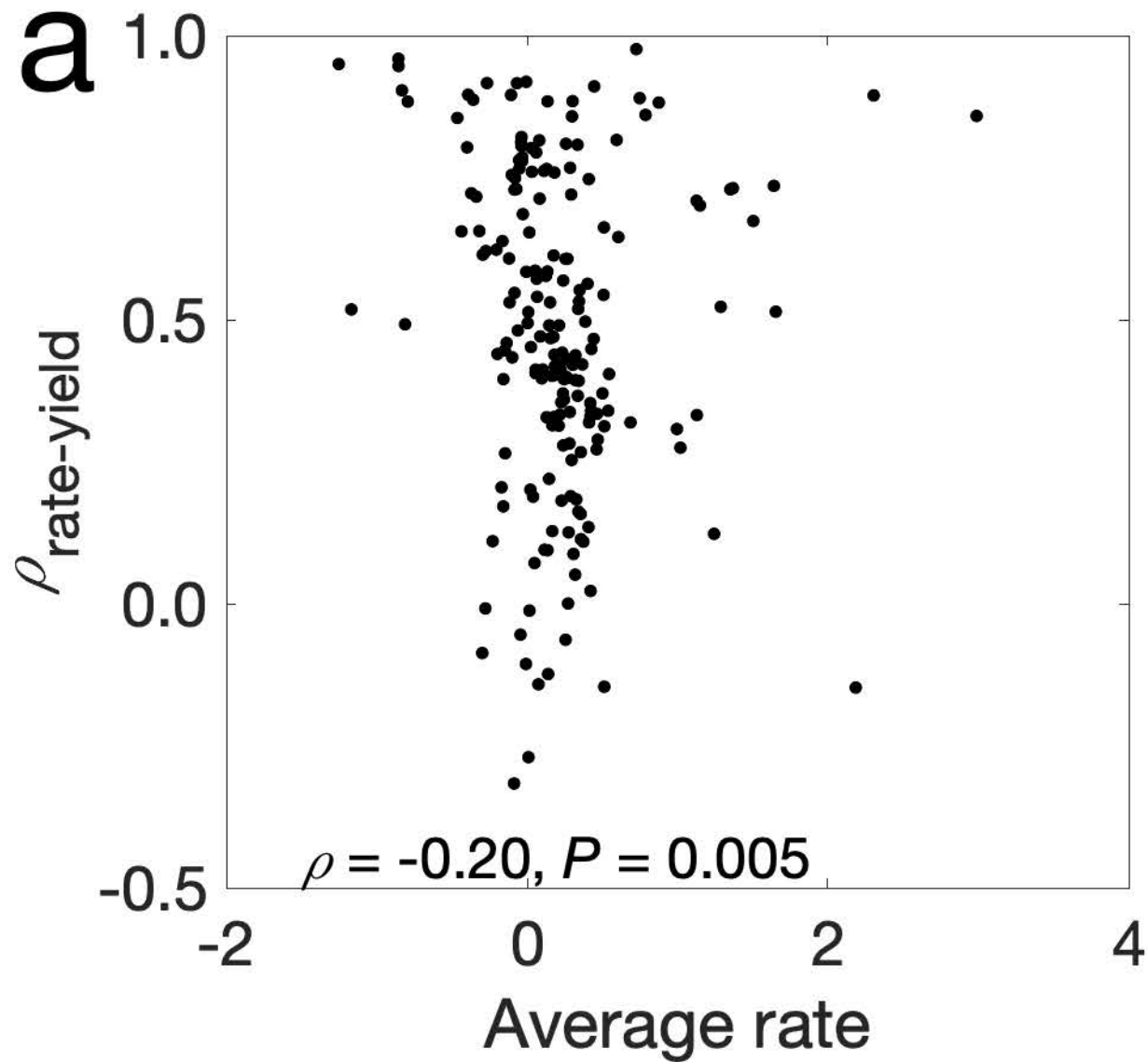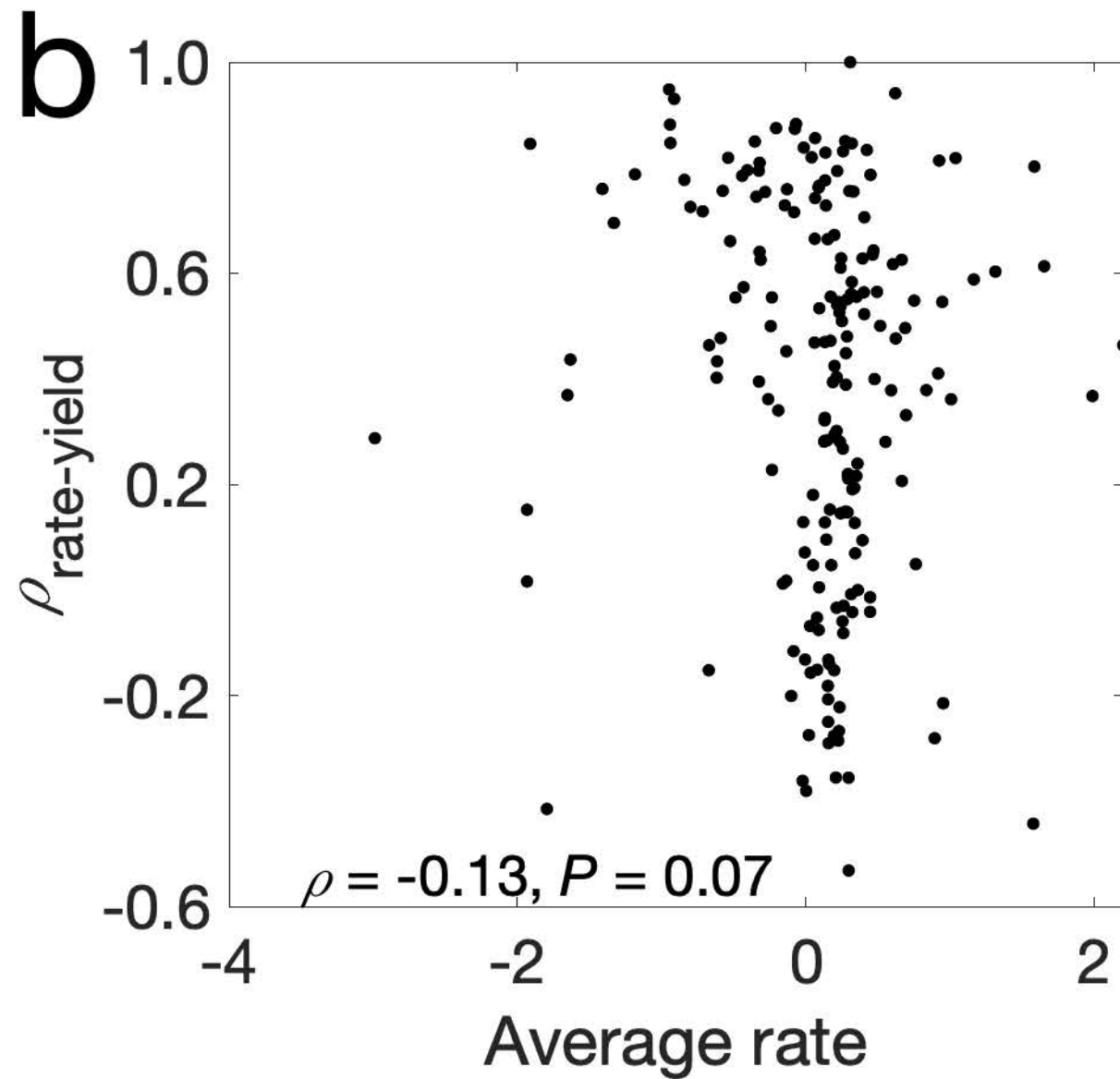

Supplement: S3 Fig — The growth rate–yield correlation in liquid media turns from positive into negative as the average rate in a medium increases in (a) S. cerevisiae and (b) S. paradoxus. Each dot represents one growth medium. Both the rate and yield estimates were from Warringer and colleagues [25]. The x-axis shows the average growth rate of all 39 measured strains in an environment, while the y-axis shows the rank correlation between rate and yield among the 39 strains in the same medium. (PDF) [file pbio.3000121.s003.pdf]
