# Supplementary material for: Environment-dependent pleiotropic effects of mutations on the maximum growth rate r and carrying capacity K of population growth
Source: PLoS Biol. 2019 Jan 25;17(1):e3000121. doi: 10.1371/journal.pbio.3000121 (PMC6364931; doi:10.1371/journal.pbio.3000121)
Supplement: S1 Table — (PDF) [file pbio.3000121.s004.pdf]

S1 Table. Variables and stresses among the nine growth media

Environment	Concentration	Type
Hydroxyurea	2.5mg/ml	Toxin
NaCl	1.4M	Cation stress
Allantoin	30mg N/L	Nitrogen source (replaces ammonium)
Caffeine	2.25mg/mL	Toxin
Galactose	2%	Carbon source (replaces glucose)
Glycine	30mg N/L	Nitrogen source (replaces ammonium)
Isoleucine	30mg N/L	Nitrogen source (replaces ammonium)
Phleomycin	2μg/ml	Toxin
Rapamycin	0.024μg/ml	Toxin
